# Crossing and selection of *Chlamydomonas reinhardtii* strains for biotechnological glycolate production

**DOI:** 10.1007/s00253-022-11933-y

**Published:** 2022-05-05

**Authors:** Antonia Schad, Sonja Rössler, Raimund Nagel, Heiko Wagner, Christian Wilhelm

**Affiliations:** 1 Department of Algal Biotechnology, Faculty of Life Science, University of Leipzig, Permoserstraße 15, D-04318 Leipzig, Germany; 2 Department of Plant Physiology, Faculty of Life Science, University of Leipzig, Johannisallee 21-23, D-04103 Leipzig, Germany

**Keywords:** Glycolate, *Chlamydomonas reinhardtii*, Algal biotechnology, Photorespiration, Sexual crossing, Strain optimization

## Abstract

**Abstract:**

As an alternative to chemical building blocks derived from algal biomass, the excretion of glycolate has been proposed. This process has been observed in green algae such as *Chlamydomonas reinhardtii* as a product of the photorespiratory pathway. Photorespiration generally occurs at low CO_2_ and high O_2_ concentrations, through the key enzyme RubisCO initiating the pathway via oxygenation of 1.5-ribulose-bisphosphate. In wild-type strains, photorespiration is usually suppressed in favour of carboxylation due to the cellular carbon concentrating mechanisms (CCMs) controlling the internal CO_2_ concentration. Additionally, newly produced glycolate is directly metabolized in the C2 cycle. Therefore, both the CCMs and the C2 cycle are the key elements which limit the glycolate production in wild-type cells. Using conventional crossing techniques, we have developed *Chlamydomonas reinhardtii* double mutants deficient in these two key pathways to direct carbon flux to glycolate excretion. Under aeration with ambient air, the double mutant D6 showed a significant and stable glycolate production when compared to the non-producing wild type. Interestingly, this mutant can act as a carbon sink by fixing atmospheric CO_2_ into glycolate without requiring any additional CO_2_ supply. Thus, the double-mutant strain D6 can be used as a photocatalyst to produce chemical building blocks and as a future platform for algal-based biotechnology.

**Key Points:**

• *Chlamydomonas reinhardtii cia5 gyd double mutants were developed by sexual crossing*

• *The double mutation eliminates the need for an inhibitor in glycolate production*

• *The strain D6 produces significant amounts of glycolate with ambient air only*

**Supplementary Information:**

The online version contains supplementary material available at 10.1007/s00253-022-11933-y.

## Introduction

The idea of using microalgal biomass as a sustainable resource for the production of high-value compounds has been intensively analysed in the last decade (Fabris et al. 2020). Although research has led to substantial technological improvements, the implementation of large-scale production units delivering chemical building blocks in a sustainable manner is still a challenge. Harvesting and refining of algal biomass are typically associated with high economic costs. Ultimately, the concept of generating bioenergy from microalgal biomass can only be economically feasible if other valuable co-products are utilized (Khanum et al. 2020). Several recent papers have also discussed this challenge and proposed ways to overcome it, such as careful selection and optimization of strains (Kong et al. 2019; Sproles et al. 2021), optimized photobioreactor design (Deprá et al. 2019; Tan et al. 2020; Legrand et al. 2021), and improved economical harvesting and processing methods (Li et al. 2020; Liber et al. 2020).

As an alternative to these techniques, decoupling biomass formation from product synthesis is another option suggested by the concept of New Green Chemistry (Wilhelm 2012). This approach is based on glycolate, a small organic carbon molecule that is naturally produced and excreted in the green microalgae *Chlamydomonas reinhardtii*. Stable long-term yield of microalgal glycolate should be possible if the corresponding cellular pathways for the production and metabolism are manipulated accordingly.

Glycolate is formed in the chloroplast stroma as part of photorespiration (Bauwe et al. 2010). Under photorespiratory conditions, i.e. high temperatures and a low CO_2_:O_2_ ratio, the balance between the carboxylase and oxygenase functions of RubisCO shifts towards the latter. Instead of CO_2_, O_2_ is captured and added to 1.5-ribulose-bisphosphate (RuBP), forming one molecule of 3-phosphoglycerate and one of 2-phosphoglycolate. While phosphoglycerate enters the Calvin cycle as usual, phosphoglycolate is hydrolysed to glycolate which is toxic to photosynthetic reactions (González-Moro et al. 1997). Thus, glycolate has to be either excreted or metabolized via the mitochondrial C2 cycle (Dellero et al. 2016). This metabolic pathway is favourable to the cell since it provides a way to recapture the fixed carbon and limit carbon losses. Unlike higher plants and cyanobacteria whose glycolate oxidase (GOX) catalyses the reaction from glycolate to glyoxylate, in most green algae, this reaction is mediated by glycolate hydrogenase (GYD) (Nakamura et al. 2005; Chauvin et al. 2008).

Stable and high glycolate excretion can be expected only if the ratio of carboxylation to oxygenation is adjusted to the value of two. If this ratio is higher, glycolate synthesis is limited by the initial reaction of the pathway. If on the other hand carboxylation is too low, the Calvin cycle will be drained, and the RubisCO reaction is limited by the concentration of the acceptor molecule. Therefore, the adjustment of the optimum oxygenation ratio is critical. However, the external ratio of CO_2_:O_2_ is modulated not only by different kinetic constants in the solubility of both gases but also by the cellular regulation of the internal CO_2_ (Iñiguez et al. 2020). *Chlamydomonas reinhardtii*, like most microalgae and cyanobacteria, controls its internal CO_2_ concentration using an elaborate system of carbon concentrating mechanisms (CCMs) (Mackinder 2018). These CCMs seem to be largely under the control of CIA5 that is thought to act as a master regulator (Fukuzawa et al. 2001; Xiang et al. 2001). As a result, the interplay between bicarbonate transporters and carbonic anhydrases maintains an increased influx of inorganic carbon across several membranes despite low external CO_2_ concentrations (Wang et al. 2015).

Due to the efficiency of the CCMs and their suppressive effect on photorespiration, long-term glycolate production in *Chlamydomonas* can only be observed if active carbon uptake is blocked. Blocking can be achieved by the addition of carbonic anhydrase inhibitors such as the sulphonamide ethoxyzolamide (EZA) (Maren 1984; Mitra et al. 2005). However, the presence of inhibitors in the harvested medium can potentially hinder the subsequent processing of glycolate. For example, heterogeneous catalysis of organic acids was found to be strongly inhibited by sulphur-containing organic compounds such as EZA (Zhang et al. 2008). In a similar vein, sulphonamides are known for their antibacterial effects (Nunes et al. 2020) which might complicate microbial fermentation of glycolate to methane (Günther et al. 2018) or the conversion into other valuable compounds. For an inhibitor-free approach, instead of using wild-type *Chlamydomonas*, glycolate can be produced by *cia5* knockout-mutants in which the CCMs are constitutively suppressed (Yun et al. 2021).

Maximizing yields of excreted glycolate also requires blocking of the photorespiratory C2 pathway so that carbon flux is directed into excretion. Günther et al. (2012) showed that inhibiting the GYD enzyme by the addition of isoniazid can increase glycolate yield. Taken together, these observations indicate that a *Chlamydomonas* double-mutant strain deficient in *GYD* and C*IA5* should allow stable glycolate production without the need for additional inhibitors.

For the generation of novel genetically modified strains, *Chlamydomonas reinhardtii* as a model organism offers a large toolbox of well-established methods (Kong et al. 2019). Random insertional mutagenesis has been used to create an extensive mutant library for research and biotechnological purposes (Li et al. 2016). More recently, new targeted approaches for gene editing such as CRISPR/cas9 (Ghribi et al. 2020) are used to create new *Chlamydomonas* strains for biotechnological applications. In addition, *Chlamydomonas* is well known for its ability for sexual reproduction (Goodenough et al. 2007); thus, new strains can also be generated by genetic crossing. The mating process can easily be induced under controlled conditions. Under nitrogen deprivation, vegetative cells differentiate into haploid gametes of two different mating types denoted *minus* (*mt −*) and *plus* (*mt* +). Sex is ultimately determined by the *MID* gene (*minus* dominance) encoded in the *MT* locus on Linkage Group VI. The presence of the *MID* gene induces the formation of *minus* gametes, while *plus* gametes only form in the absence of the gene (Ferris and Goodenough 1997). While most genes of the *MT* locus are found in both mating types, *FUS1* is one of the few genes unique to the *MT* + locus. It encodes for a *plus*-specific glycoprotein that is necessary for gamete fusion (Ferris et al. 1996; Misamore et al. 2003). After a maturation period of a few days, the newly formed zygote undergoes a reduction division resulting in a tetrad of four haploid cells, each with a different genetic background.

So far, sexual reproduction in *Chlamydomonas* has been studied mainly in the context of basic research, while less attention has been given to its potential use for breeding new strains of biotechnological interest. Backcrossing mutants of interest with wild-type strains is usually done to optimize key features in the second generation, such as mating type or presence of a cell wall. The advantage of such a crossing strategy is that the optimization process can be based on wild-type cells selected according to the highest physiological activity, growth potential, and mechanical robustness. These features are essential for strains designed for biotechnological applications. The crossing concept aims to preserve the best physiological functionality in mutants with specific gene modifications according to biotechnological needs. In this study, we used the method of crossing high-performance wild types of *Chlamydomonas reinhardtii* with mutants lacking the function of the genes for glycolate-metabolizing GYD and the CCM master regulator protein CIA5. Thus, both mutations are combined within one strain to optimize the cellular metabolism towards glycolate production. The selection of strains in the crossbreeding process was based on the optimal physiological strain performance of each strain, e.g. highest photosynthetic rates. Thus, the designed double mutants should have the best physiological performance and robustness for industrial glycolate production. These double mutants are then an ideal starting point for further process optimization for glycolate production.

## Methods

### Algal strains and culture media

For the development of new *cia5 gyd* double mutants, several wild-type and mutant strains of *Chlamydomonas reinhardtii* were subjected to a conventional crossing procedure. In the initial *gyd* mutant LMJ.SG0182.017965, the *GYD* gene is disrupted via the insertion of a drug-resistance gene cassette (Zhang et al. 2014). The *cia5* mutant CC-2702 harbours a point mutation in the C*IA5* gene and is defective in its induction of CCMs (Fukuzawa et al. 2001; Xiang et al. 2001). For both mutants, two wild-type strains CC-410 and 11-32b with opposing mating types (*mt − *and *mt* + respectively) were chosen as crossing partners. All strains were obtained from the Chlamydomonas Resource Center (Minnesota), except for the wild type 11-32b (Culture Collection of Algae, Göttingen, Germany). The physiologically optimized strain CC-5759 D6 *cia5*/*gyd* was deposited at the Chlamydomonas Resource Center and will be referred to as D6 in this study.

For the maintenance of the strains, we used TRIS acetate phosphate media (TAP) (Gorman and Levine 1965) modified with 25 µM Fe-EDTA and trace elements from Bold’s Basal Medium (BBM) (Bischoff and Bold 1963). For TRIS-minimal medium (TP), the acetate was omitted, and the medium was adjusted to pH 7.0 with HCl. Both TAP and TP agar plates were prepared by adding 2% (w/v) agar. For long-term glycolate excretion experiments, TRIS buffer was substituted by 20 mM MES (2-(N-morpholino)ethanesulphonic acid).

### Crossing of Chlamydomonas strains

**Fig. 1:**
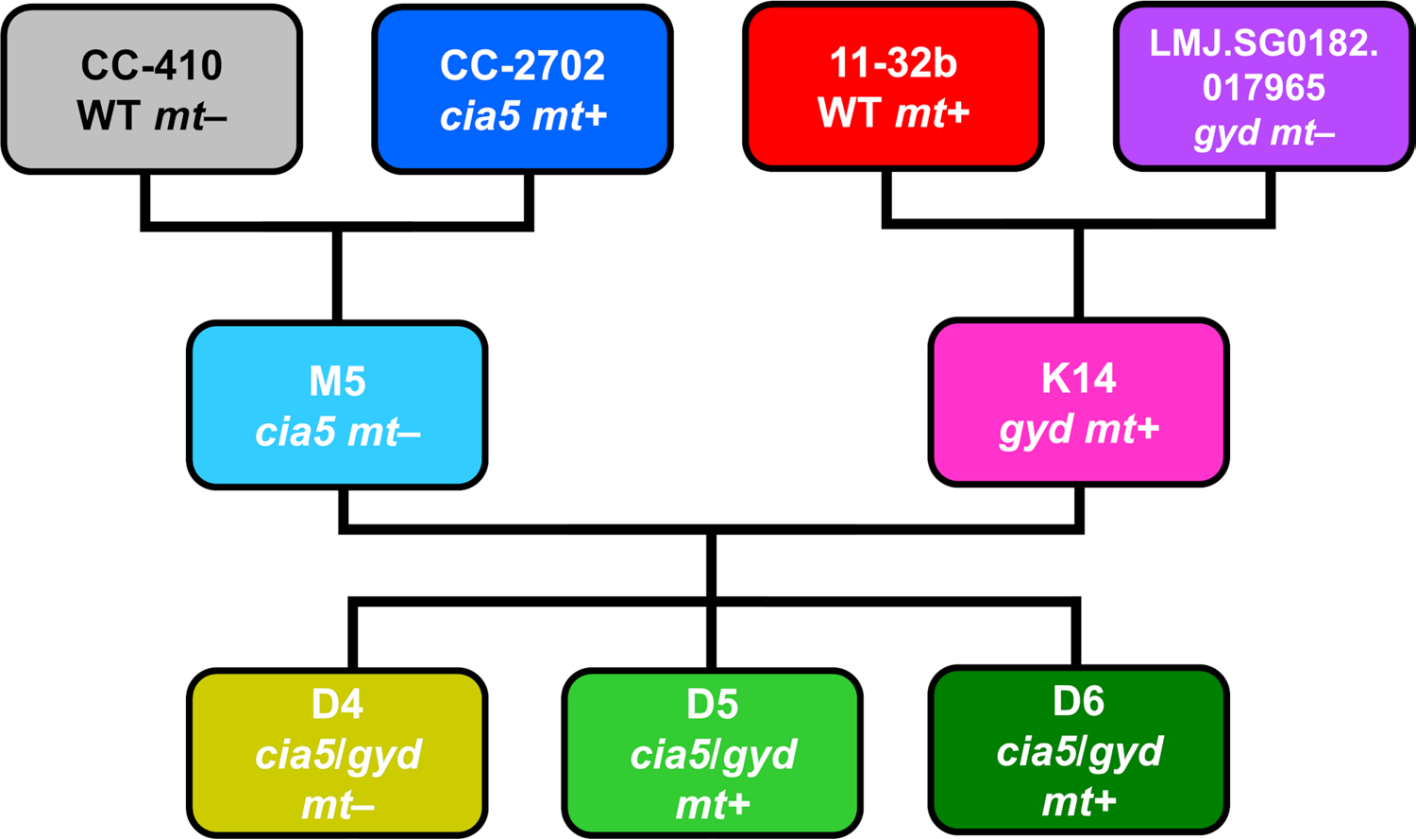
Crossing strategy for the development of *Chlamydomonas reinhardtii* double mutants. First, wild-type (WT) strains CC-410 and 11-32b were crossed with the initial *cia5* and *gyd* mutants respectively, resulting in several new second-generation strains. Out of these, M5 and K14 were chosen for the second crossing step from which *cia5 gyd* double mutants emerged in the third generation

The breeding strategy followed a two-step crossing plan with an intermediary screening procedure to select the most suitable strains for the next crossing step (Fig. 1). First, the *gyd* and the *cia5* mutant were crossed with wild-type strains 11-32b and CC-410 respectively, yielding a second generation of single mutants (K- and M-lines). All second-generation strains were subsequently screened for selected physiological traits (growth rates, photosynthetic capacity, and short-term glycolate production rate) as described below. Mating types were also determined. Strains with the most promising results for the selected traits were crossed again to create a third generation of *cia5 gyd* double mutants.

For the crossing of two strains, a protocol by Jiang and Stern (2009) was used. Briefly, healthy strains were maintained on nitrate-free TAP agar plates for four days. On the day of mating, strains were suspended in 10 ml of sterile distilled water to induce flagella synthesis. After 2 h of gently stirring the cultures at a light intensity of 1200 µmol photons m^−2^ s^−1^, strains were mixed in equal concentrations to a final number of 10^7^ cells ml^−1^. Again, after 2 h, 2 ml of the mating mixture was plated in four spots on 4% (w/v) nitrate-free TAP agar and left to dry. The plates were kept in the dark for 1–2 weeks to induce the formation of zygospores. To obtain new single clone cultures from zygotes, tetrad isolation and separation followed as described in the protocol (Jiang and Stern 2009). After transferring the zygotes to fresh agar plates, accidentally transferred vegetative cells were killed by exposing the plates to chloroform vapour for 30 s. Thus, any newly emerging colonies should be attributed to zygote germination and tetrad multiplication only.

### Detection of mutated GYD gene and the MID and FUS1 genes

To confirm successful crossing between two strains, the progeny was screened not only for mutant genes but wild-type genes as well. This was to ensure that selected colonies originated indeed from sexual reproduction and not from asexual division of the original mutant parents. Wild-type genetic material in the progeny was detected by determining the mating type via the presence of sex-specific *MID* or *FUS1* genes respectively.

*MID* and *FUS1* genes as well as the mutated *GYD* gene could be detected by polymerase chain reaction (colony PCR). Genomic DNA was extracted from a pellet of each strain resuspended from TAP agar plates into 50 µl of 10 mM NaEDTA. The samples were lysed in a thermocycler at 100 °C for 5 min. After 2 min of centrifugation at 12,000 g, 1.5 µl of the supernatant was used in the PCR.

Mating type determination based on *MID* and *FUS1* genes was conducted according to a protocol by Werner and Mergenhagen (1998) using the primer sequences stated therein. Primers for *GYD* amplification were *GYD* 5'UTR fwd1 and *GYD* Jonikas rev1 (Table S1). The genomic DNA template was diluted to concentrations between 50 and 100 ng ml^−1^. Two microliters of genomic DNA template was amplified in a 25 µl-reaction containing 0.625 units OptiTaq Polymerase (Roboklon, Berlin), 200 µM deoxynucleotide triphosphates, each of the respective primers at 0.6 µM, 5 µl of the provided buffer, and 2 µl dimethyl sulphoxide (DMSO). Reaction mixtures were incubated in a PCR thermocycler (peqSTAR, peqlab, VWR International, Germany) with cycling parameters for reactions with the *MID/FUS1*-specific primers as follows: 3 min at 95 °C, 35 cycles of 30 s at 95 °C, 30 s at 52 °C, 60 s at 72 °C; followed by a final extension of 7 min at 72 °C. For the *GYD*-specific primers, the cycling parameters were modified to 5 min at 95 °C, 35 cycles of 30 s at 95 °C, 30 s at 58 °C, 4 min at 64 °C, and a final extension at 72 °C for 7 min.

The products were run on a 1.5% (w/v) agarose gel. DNA staining was achieved by adding 5 µl ethidium bromide to the gel cassette prior to applying the samples.

### Detection of mutated CIA5 gene

For an initial quick screening of the mutated *CIA5* gene in the second- and third-generation strains, we searched for strains with a high-CO_2_-requiring phenotype, as *cia5* mutants are strongly inhibited in phototrophic growth (Van et al. 2001). Growing cultures from freshly plated TAP agar plates were streaked onto 2% (w/v) agar plates with and without an additional inorganic carbon source in form of acetate (TAP and TP plates respectively). The plates were kept at ambient CO_2_ concentration (0.04% CO_2_ in air (v/v)) and low light intensities of 15 µmol photons m^−2^ s^−1^ for 12 days.

Additionally, for the double mutant strains D4, D5, and D6, the *CIA5* mutation was verified at the nucleotide level. TAP-grown cultures were centrifuged at 8,000 g for 5 min. Genomic DNA was isolated using the Plant Genomic DNA Mini Kit (Geneaid). A fragment of 820 bases around the affected base was amplified with primers for *CIA5* amplification (Table S1) and Q5® High-Fidelity DNA Polymerase (New England Biolabs, Ipswich, USA). In total, 0.5 µl of genomic DNA template was added to a 25 µl-reaction containing 0.5 units Q5® Polymerase, 200 µM deoxynucleotide triphosphates, each of the respective primers at 0.5 µM, 5 µl 5X Q5 High GC Enhancer and 5 µl of the provided buffer. Cycling parameters were as given in the manufacturer’s instructions with an annealing temperature of 58 °C. PCR fragments were separated on an agarose gel and stained with ethidium bromide as described above. After excising the desired fragments from the gel, DNA was extracted using the GeneJET Gel extraction kit (Thermo Fisher Scientific, USA). Sequencing of the samples in both directions, using the above-described primers, was done by Eurofins Genomics (Germany).

### Detection of a functional cell wall for gyd mutants

Since the initial insertional *gyd* mutant used for the crossing strategy was based on a wall-less *cw* strain (Zhang et al. 2014), the progeny was tested for a functional, stable cell wall. Five hundred microliters of 1% (v/v) Triton X-100 was added to an equal volume of cells suspended in TAP medium. For a control measurement, 500 µl of distilled water was used instead. After 10 min of incubation, cell concentrations in both samples were determined using an automatic cell counter (Z2, Coulter Electronics Inc., Miami, USA) and the percentage of cells lysed by the detergent was calculated.

### Cultivation conditions for physiological measurements

Batch cultures of each strain were placed in a photobioreactor (MC 1000, Photon Systems Instruments) comprising 8 independent 70-ml vessels. All cultures were illuminated with 150 µmol photons m^−2^ s^−1^ with a day length of 14 h. Aeration conditions varied depending on the experimental setup (Table 1). Preparatory cultures were cultivated with A1 aeration condition (5% CO_2_ in ambient air (v/v)) before the start of the experiment.

**Table 1:** Aeration conditions for experimental setups. For condition A2, aeration with ambient air was used. For all other conditions, ambient air was supplemented with additional CO_2_ and/or O_2_ (v/v)

Condition		Composition (v/v)	CO_2:_O_2_ ratio
A1	5% CO_2_	20% O_2_, 5% CO_2_	0.25
A2	Ambient air	20% O_2_, 0.04% CO_2_	0.002
A3	Elevated O_2_ and CO_2_	40% O_2_, 0.08% CO_2_	0.002
A4	Elevated O_2_ and CO_2_	30% O_2_, 0.2% CO_2_	0.007

### Determination of chlorophyll content and cell concentration

For chlorophyll *a* (Chl *a*) determination, 2–6 ml of algal samples were collected on a cellulose filter (MN 85/70, 25 mm, Macherey–Nagel, Düren, Germany). Two millilitres of 80% (v/v) acetone and glass beads (0.25–0.5 mm and 0.75–1 mm; 3:1) were added, then the cells were broken in a homogenizer (Precellys Evolution, Bertin Technologies, France). After centrifugation, absorbance at 664 and 647 nm was measured with a spectrometer (U-2000 Hitachi). Chl *a* concentration was calculated according to Ziegler and Egle (1965).

Cell concentration inside the samples was determined with an automatic cell counter (Z2, Coulter Electronics Inc., Miami, USA).

### Growth rates in high CO_2_conditions

To measure phototrophic growth rates, acetate-free TP medium was used to dilute preparatory cultures to a starting concentration of 5 × 10^4^ cells per vessel. Cultures were gassed with 5% CO_2_ in ambient air (A1). Samples were taken twice a day for at least four consecutive days. Growth rate μ [d^−1^] was then calculated as the slope of the linear least square regression of the natural logarithm of the increase in cell number or chlorophyll content against time (Fanesi et al. 2016).

### Measurements of photosynthetic oxygen production

Cells of each strain were cultivated semi-continuously in the photobioreactor vessels at a pigment content of 2–2.5 mg Chl *a* l^−1^ with A1 aeration and the reactor setup described above. Oxygen-based photosynthesis and respiration rates were measured using a so-called light pipette as described in detail in Wagner et al. (2006). This device was equipped with a special cuvette (Topgallant LLC, Salt Lake City, UT, USA) that was combined with a Clark-type electrode (MI730, Microelectrodes Inc., Bedford, NH, USA). A culture sample of 4 ml was filled into the cuvette. Photosynthesis-irradiance curves were measured for 50 min using a computer-controlled light regime with six increasing light intensities of 4 min each, alternated with dark phases of the same length. Gross oxygen production was calculated by correcting the net oxygen production rate during light phases for the corresponding dark respiration in the preceding dark phases. Photosynthesis-irradiance curves (Figure S1) were then fitted using the algorithm of Eilers and Peeters (1988) to determine the maximum photosynthetic rate (P_max_).

### Determination of short-term glycolate production rates with EZA

To induce glycolate production, the aeration of preparatory cultures was switched from condition A1 to A3 at a pigment content of 2–2.5 mg Chl *a* l^−1^. At the same time, a final concentration of 50 µM ethoxyzolamide (EZA) dissolved in 50% (v/v) ethanol was added. Samples were collected every 2 h and algal cells were removed by centrifugation. The supernatant was sterile-filtered (0.2 µm filter; Roth, Karlsruhe, Germany), freeze-dried, and stored at − 20 °C until further use. The quantitative determination of glycolate concentration was based on a colorimetric method using 2.7-dihydroxynaphthalene in sulphuric acid (Calkins 1943). The dried samples were resuspended in distilled water and 1.5 ml of the staining reagent was added to 50 μl of the sample and incubated in a water bath at 100 °C for 20 min. Immediately afterwards, the reaction was stopped on ice and the absorbance of the samples was recorded at 540 nm (Specord M250, Zeiss, Jena, Germany). Glycolate standards for calibration were measured in spent medium supernatant harvested at the start of the experiment.

### Long-term glycolate excretion in the D6 cia5 gyd double mutant

To check whether the double mutation can effectively substitute for the effects of the inhibitor EZA, glycolate production in the new strain D6 and wild type was studied under different aeration conditions. When the preparatory cultures reached biomass of 2 mg Chl *a* l^−1^, a final concentration of 50 µM EZA was added, and the aeration was switched from A1 to either A2 (ambient air) or A4 (O_2_ and CO_2_ rich conditions). Glycolate samples were taken as described above. For these long-term experiments, glycolate detection was performed by HPLC with a Waters liquid chromatography system, which consisted of a 600-MS system controller, an autosampler 717, and a 966-photodiode array detector. A Eurosphere II 100-S C18 column was used, fitted with a pre-column to retain impurities. The column temperature was kept at 15 °C. Isocratic separation was performed at a flow rate of 0.8 ml min^−1^ with 14 µM phosphoric acid as eluent. Glycolate was detected by UV absorption at 210 nm and calibrated with external standards. The concentration in the sample was then calculated from the integrated area under the absorbance curve using Empower Pro 2 software (Waters, Eschborn, Germany).

### Statistical analysis

For physiological parameters e.g. growth rates, oxygen production, and glycolate production rates, values of three or more replicates were averaged and standard deviation was calculated. One-way ANOVA was used to determine statistical differences, followed by a post hoc Bonferroni test. A *p* value less than 0.05 was considered statistically significant. To examine the correlation strength between physiological parameters, Pearson’s correlation coefficient was used. Statistical analysis was performed using GraphPad Prism version 5.03 (GraphPad Software, La Jolla, CA).

## Results

### Cia5 mutants showed impaired growth on air level concentrations of CO_2_

**Fig. 2:**
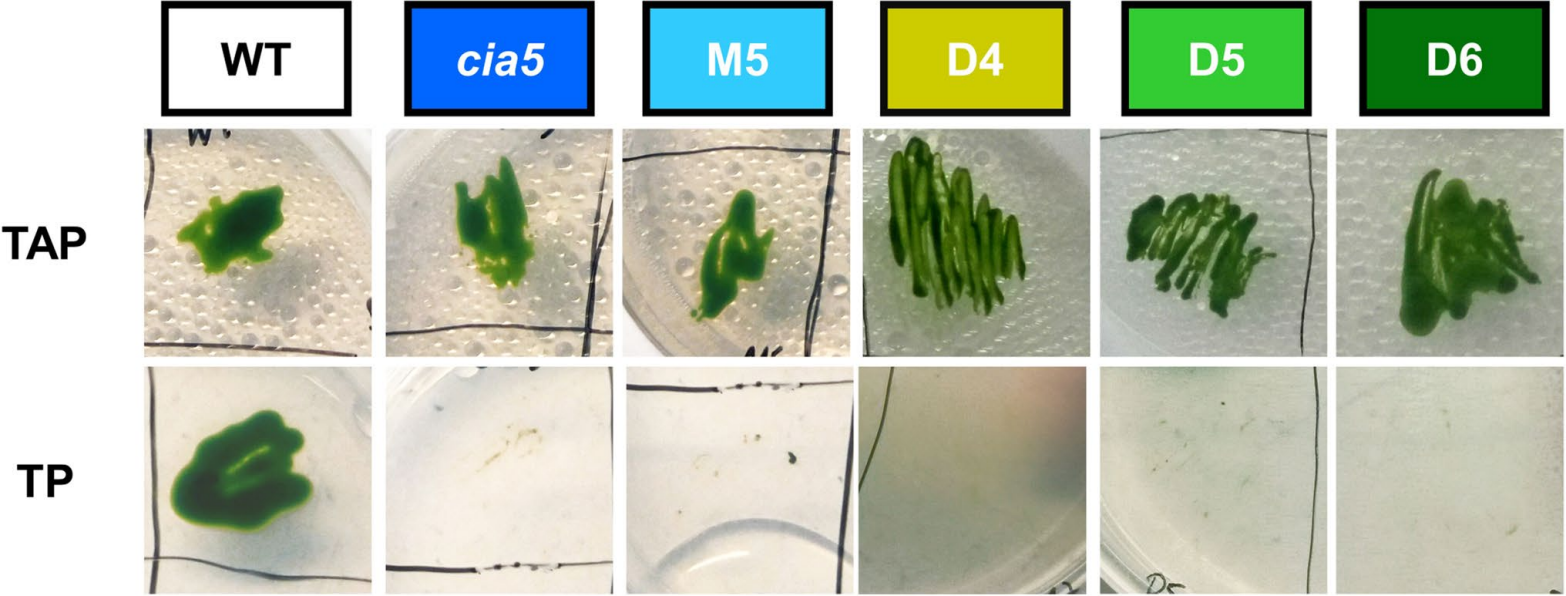
Growth of *Chlamydomonas reinhardtii* wild-type (WT) and mutant strains on plates on medium with the addition of acetate (TAP) and on acetate-free medium (TP)

After crossing the initial *cia5* mutant with wild-type strain CC-410, 10 newly isolated tetrads showed successful colony growth. To identify strains with a disruptive *CIA5* gene, colony growth in response to low and high carbon supply was tested. Crossing products and parent strains were propagated on acetate-containing plates (TAP) and TRIS minimal medium (TP) at ambient CO_2_ (0.04%). While the initial *cia5* mutant grew well on TAP plates, its growth was impaired on acetate-free TP medium (Fig. 2). This phenotype is expected for strains with defective CCMs and is frequently used as an indication for strains with impaired carbon acquisition which require CO_2_ concentrations above air level to survive (Van et al. 2001).

The wild type on the other hand was able to grow both with and without the addition of acetate. Out of 10 WT x *cia5* crossing products, 4 showed impaired growth on acetate-free medium similar to the *cia5* parent, suggesting they also carried the defective *CIA5* gene. Among these, strain M5 was used for further strain development.

**Fig. 3:**
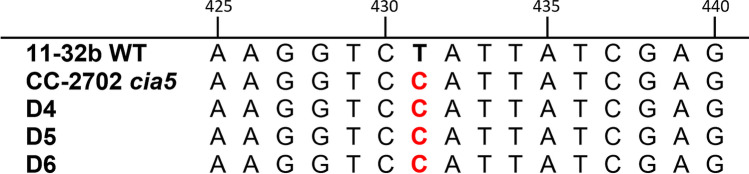
Sequence comparison of *CIA5* amplification products in *Chlamydomonas* wild-type and mutant strains. The disrupted *CIA5* gene features a point mutation from T to C at position 431 (marked red)

In the third generation, the growth test identified three double mutants D4–D6. Additionally, the defect in *CIA5* was confirmed for these strains by sequencing the gene locus (Fig. 3). D4 –D6 as well as the original *cia5* mutant featured a T to C point mutation at position 431. This substitution results in the replacement of the His-54 by Tyr in one of the two zinc-finger domains of the CIA5 protein, disrupting its function as a regulator of CCM induction (Fukuzawa et al. 2001; Xiang et al. 2001).

### A PCR screening identified strains with a disrupted GYD gene

**Fig. 4:**
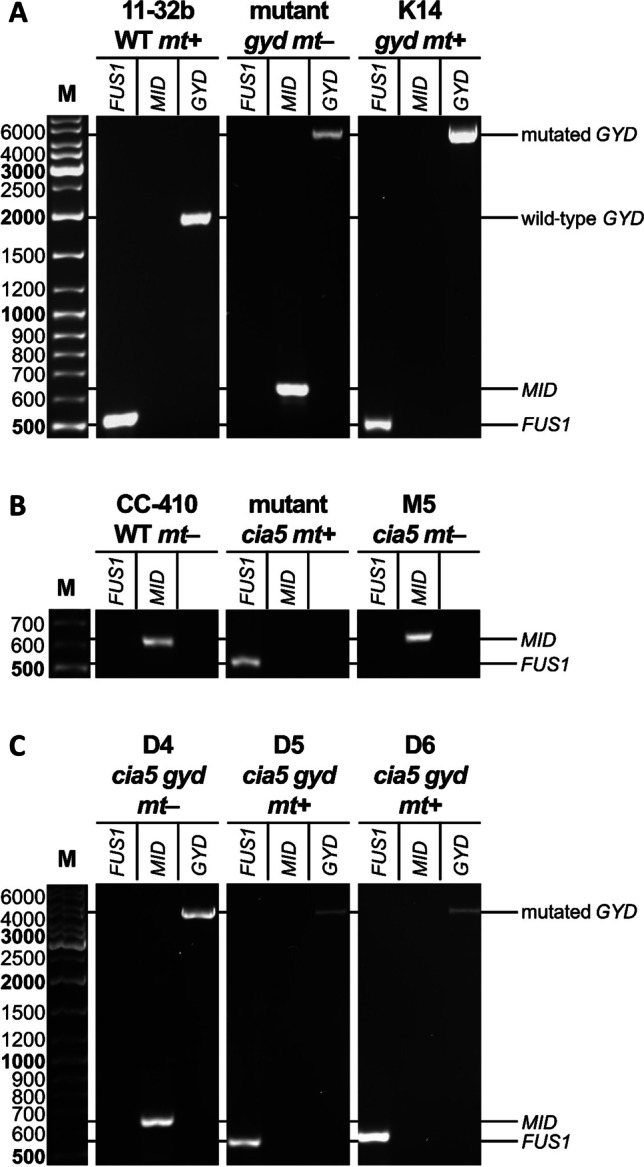
Genotype determination of *Chlamydomonas* strains to identify mating type and *gyd* mutants. **A** Crossing product K14 and parent strains for the cross of 11-32b WT *mt* + and the original *gyd* mutant *mt‒.* **B** Crossing product M5 and parent strains for the cross of CC-410 WT *mt‒* and the original *cia5* mutant *mt* + . **C** Crossing products D4-D6 from the cross of K14 *gyd* x M5 *cia5*. All strains were combined with primers specific for *FUS1* and *MID* to determine mating type. *GYD*-specific primers were used to determine wild-type and mutant *GYD* gene respectively

For the cross of the initial *gyd* mutant with wild-type 11-32b, 22 isolated crossing products were screened for those carrying the mutated *GYD* gene (Fig. 4A). The presence of the mutation was detected via PCR. Unlike the amplification product of the wild-type *GYD* gene with an expected size of about 1800 bp, the mutated *GYD* yielded a much larger product of ~ 4500 bp. Its larger size was due to a 2260 bp insertion cassette that disrupts the gene function in the mutant (Zhang et al. 2014). Both of the respective amplification products were easily distinguishable after separation on an agarose gel. Out of 22 isolated second-generation crossing products, 12 carried the defective *GYD* gene, including the strain K14 (Fig. 4A). In the third generation, the same method was used to detect the disrupted *GYD* gene in the three *gyd cia5* double mutants, D4–D6 (Fig. 4C).

The second-generation strain K14 was also tested for the presence of a functional cell wall. The results showed that the new strain K14 was as insensitive to the chemical detergent as its wild-type parent (Figure S2). With both strains, less than 10% of the cells subjected to the detergent were lost during cell count, suggesting that the cell wall protected most of the cells from lysis. In contrast, the application of the detergent to a wall-less control strain resulted in lysis of > 99% of the cells.

For the *cia5* M-lines, such verification was not necessary as the initial *cia5* mutant is not known to have a deficient cell wall (Katzman et al. 1994).

### The presence of FUS1 or MID indicated the mating type of new strains

Due to the crossing procedure, it was not directly possible to distinguish whether K- and M-colonies carrying the desired mutations originated from a sexual mating or just from unmated vegetative clones of the mutant parent. Descendants resulting from fused gametes should carry genetic material from the mutant parent and the wild type. For this purpose, the sex-linked genes from the wild-type parent (*FUS1* and *MID*) were used as unequivocal evidence of a successful cross. If a mutant descendant has the same mating type as its wild-type parent, this can be strictly attributed to a sexual crossing event and rules out a simple asexual division. Consequently, in the second generation, mating type determination was used to identify haploid descendants among the crossing products that resulted from a sexual cross.

Both mating types could be easily distinguished by the different specific genes present in the cells. All first-generation parent strains showed the expected PCR amplification products corresponding to their known mating types (Fig. 4A + B). Thus, for the wild-type 11-32b and CC-2702 *cia5*, the 516 bp amplification product of *FUS1*, the gene that is unique for the *mt* + type, was found. In contrast, *MID* was not present in the locus. For the wild-type strain CC-410 and the original *gyd* mutant, on the other hand, *MID* was detected but not *FUS1*, confirming the mating type *minus* (*mt‒*). For second-generation strains, successful sexual crossing was only assumed if they featured the same mating type as their wild-type parent, representing the transfer of wild-type genetic material. Only those mutant strains were taken into consideration as suitable candidates for the second crossing step. Altogether, 5 out of the 12 K-strains for which a defective *GYD* gene had been detected beforehand also carried the *FUS1* gene, among them the strain K14 *gyd*. Respectively, 6 out of 10 WT x *cia5* crossing products (M-strains) inherited a *minus* mating type (*mt‒*) from their wild-type parent, such as the strain M5 *cia5*. Crossing products that showed both *FUS1* and *MID* bands and were thus likely to be diploid were discarded as candidates for the second crossing.

In the third generation, strains with both *mt‒* (presence of *MID* gene, e.g. strain D4) and *mt* + (presence of *FUS1*, e.g. strain D5 and D6) were identified (Fig. 4C).

### Strains of all three different generations were screened for relevant physiological traits

**Fig. 5:**
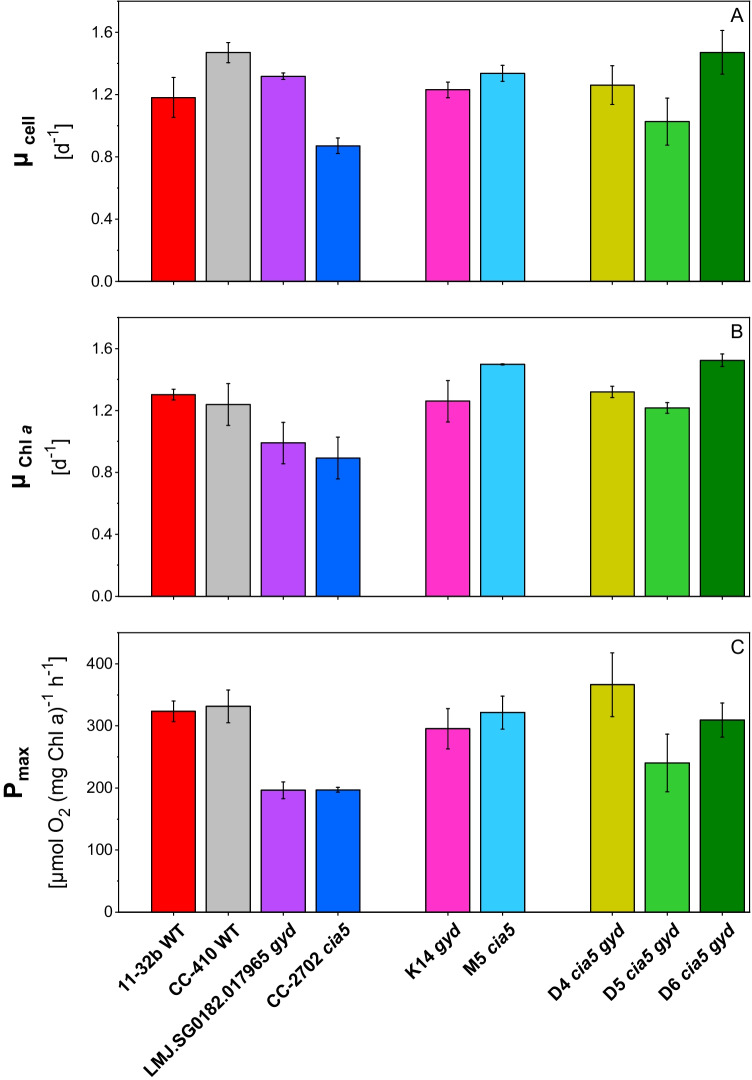
Comparison of several physiological traits among *Chlamydomonas reinhardtii* wild types and different generations of mutants. Strains were cultivated at 5% CO_2_ in ambient air and examined for their daily growth rate µ based on **A** cell number and **B** chlorophyll content. Maximum photosynthetic capacity P_max_ was estimated as well (**C**). Shown are mean values for each strain (*n* ≥ 3). Errors bars indicate standard deviation

Following both of the crossing steps, several physiological traits were monitored to test the performance of the new mutant strains compared to their parental strains. Initially, in the first generation, growth rates based on Chl *a* content (µ_Chl *a*_) and cell number (µ_cell_) were high in the wild types, but comparatively low in the *cia5* mutant (Fig. 5A + B). However, after crossing, the growth rate µ_cell_ of the second-generation strain M5 carrying the *CIA5* mutation reached a value of 1.3, which is 40% higher compared to the initial *cia5* parent (p < 0.001). A similar significant increase was observed for Chl *a*–based growth rates with a value of µ_Chl *a*_ = 1.5 for M5 (*p* < 0.001). Hence, the growth rates of M5 were more comparable to its wild-type parent CC-410.

In the first generation, the *gyd* mutant had a high cell number–based growth rate with µ_cell_ = 1.3 while the daily increase in chlorophyll was lower with µ_Chl *a*_ = 1.0. The second-generation *gyd* mutant K14 had a similar high µ_cell_; however, µ_Chl *a*_ increased to 1.3. This altered ratio between µ_Chl *a*_/µ_cell_ points to an increase in cellular chlorophyll content between generations.

Crossing M5 and K14 yielded a third generation, in which the increased growth rates of the second generation could be maintained. With a daily growth rate of µ_cell_ = 1.47 and µ_Chl *a*_ = 1.52, the double mutant D6 in particular was as efficient as the wild-type CC-410.

The observed growth rates were in line with the maximum photosynthetic capacity (P_max_) of the different generations (Fig. 5C). While P_max_ was high in wild-type cells with oxygen production rates from 320 to 330 µmol O_2_ (mg Chl *a*)^−1^ h^−1^, the initial *gyd* and *cia5* mutant only reached a P_max_ of about 197 µmol O_2_ (mg Chl *a*)^−1^ h^−1^. After crossing, P_max_ of the second-generation strains increased significantly up to 295 and 321 µmol O_2_ (mg Chl *a*)^−1^ h^−1^ for M5 and K14 respectively (*p* < 0.001). The third-generation strain D6 reached a maximum production rate of about 310 µmol O_2_ (mg Chl *a*)^−1^ h^−1^ and was thus comparable to the wild type in its photosynthetic efficiency. P_max_ of D4 even surpassed the wild-type rates, with 366 µmol O_2_ (mg Chl *a*)^−1^ h^−1^. A cross-generational correlation was found for P_max_ and µ_Chl *a*_ with Pearson’s test (*ρ* = 0.82, *p* < 0.01; Figure S3).

**Fig. 6:**
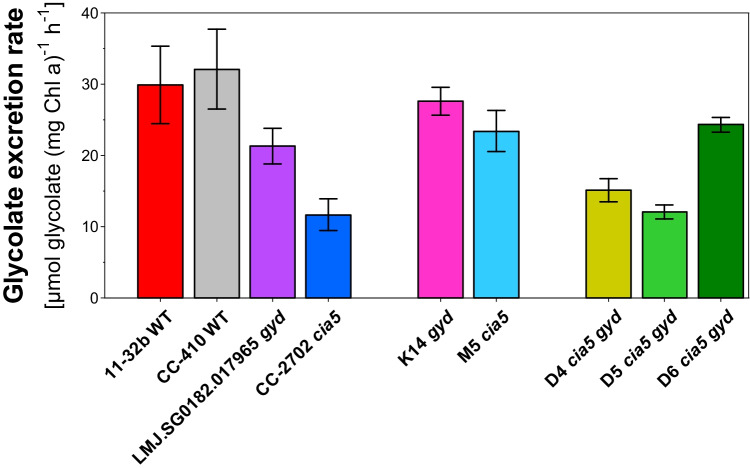
Comparison of short-term glycolate production rates among *Chlamydomonas reinhardtii* wild types and different generations of mutants. Glycolate excretion rates were measured over the course of 6 h at 40% (v/v) O_2_, 0.08% (v/v) CO_2_ within the presence of a carbonic anhydrase inhibitor. Shown are mean values for each strain (*n* ≥ 3). Error bars indicate standard deviation

To assess the potential for increased glycolate excretion of the new strains in comparison to the wild type, short-term glycolate production rates of all strains were measured at elevated O_2_ (aeration condition A3). In strains with a functional CCM (i.e. featuring the wild type *CIA5* gene), no glycolate excretion can be observed without the inhibitor (Figure S4). Thus, to screen for strains with an optimized physiological background that allows efficient glycolate excretion, comparative measurements were conducted in the presence of the inhibitor. The highest short-term glycolate production rates were found for both wild-type strains (Fig. 6) with rates of > 30 µmol glycolate (Chl *a*)^−1^ h^−1^. These values reflect the physiological potential that can be achieved by CCM suppression and should thus be matched by the double mutants. Initially, in the first generation, only 69% of this potential could be reached by the *gyd* mutant and 38% by *cia5*. These findings were in line with the low P_max_ values of these strains, entailing a lower carbon fixation capacity. After crossing, the second-generation strains showed a clear increase in glycolate production rates when compared to their mutant parents. The maximum production rate in the strain M5 *cia5* doubled from 11.7 to 23.4 µmol glycolate (Chl *a*)^−1^ h^−1^, while the rate of K14 *gyd* increased by 30%.

However, such high production rates could not be maintained in the third-generation strains, except for strain D6. While the excretion rates for D4 and D5 both remained < 15 µmol glycolate (mg Chl *a*)^−1^ h^−1^, D6 showed a higher production rate of 24 µmol glycolate (mg Chl *a*)^−1^ h^−1^.

### The D6 cia5 gyd strain produced glycolate with ambient air and without inhibitor

**Fig. 7:**
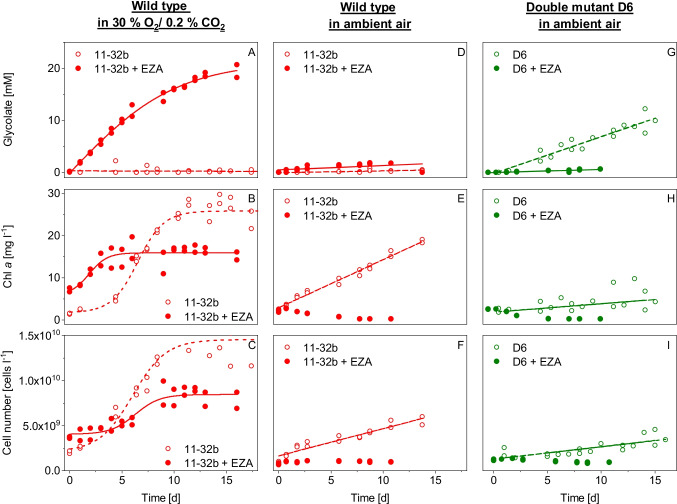
Long-term glycolate production of wild type 11-32b and D6 mutant. Shown are two replicates for each strain in either inhibitor-free conditions (open dots) or with the addition of a carbonic anhydrase inhibitor (closed dots). Cultures were aerated with either 30% (v/v) O_2_, 0.2% (v/v) CO_2_ (A4 condition) or ambient air (A2 condition). For a period of more than 14 days, glycolate concentration, Chl *a* content, and cell numbers were determined. Hyperbolic and linear fits were used for the data to illustrate culture growth and glycolate production (plain and dotted lines)

To evaluate the *cia5 gyd* double mutant as an alternative glycolate production platform to the established wild-type system, long-term glycolate production of each strain was monitored for several days. The wild type accumulated glycolate to concentrations of 19.5 mM within 16 days with adjusted aeration (A4) and the addition of the inhibitor (Fig. 7A), resulting in a daily production rate of 1.2 mM glycolate day^−1^. After an initial growth phase in the first few days, the biomass concentration inside the reactor stabilized at around 16 mg Chl *a* l^−1^ (Fig. 7B) and 8.5 × 10^9^ cells l^−1^ (Fig. 7C) respectively.

As expected, these production rates could only be achieved in wild types by adding a carbonic anhydrase inhibitor, as no glycolate was found inside the medium without the inhibitor. Instead, the biomass increased under these experimental conditions, reaching saturation at about 26 mg Chl *a* l^−1^ (Fig. 7B) and much higher cell concentrations of about 1.5 × 10^10^ cells l^−1^ (Fig. 7C).

However, wild-type cells did not produce significant amounts of glycolate when aerated with ambient air at 0.04% CO_2_ under both conditions, with and without the carbonic anhydrase inhibitor (Fig. 7D). Without the inhibitor, these cultures were growing slower than those aerated with additional CO_2_, but at steady daily rates of µ_Chl *a*_ = 0.14 and µ_Cell_ = 0.10 (Fig. 7E + F). However, in the presence of the inhibitor, cells immediately bleached, and the culture collapsed.

The preceding double mutant screening revealed D6 as the strain with the highest potential for glycolate production. The strain’s performance was, therefore, further tested in long-term experiments. Although short-term experiments showed these cells produced glycolate under high oxygen concentrations (A3), we were unable to maintain cultures for longer periods under these conditions previously used for the wild type. Within a few days under elevated O_2_, the cells started to bleach and the culture died (Figure S5). This condition therefore is obviously unsuitable for establishing an efficient long-term glycolate production system. Instead, we found that D6 showed efficient glycolate excretion without addition of EZA when aerated with ambient air (A2). Within 16 days, glycolate accumulated up to 10 mM, resulting in production rates of 0.63 mM glycolate day^−1^ (Fig. 7G). Biomass growth based on cell numbers and Chl *a* concentrations was limited under these glycolate production conditions, with low daily rates of µ_Chl *a*_ = 0.06 and µ_Cell_ = 0.05 (Fig. 7H + I). Under ambient air, these cultures could be kept stable for several weeks without a decrease in production efficiency. In contrast, when EZA was added to the D6 strain, the culture collapsed and no glycolate accumulation was detectable.

## Discussion

### A physiological screening revealed the positive impact of the breeding strategy

In previous publications, the green alga *Chlamydomonas reinhardtii* was established as a continuous glycolate production platform (Taubert et al. 2019). Wild-type cells could be kept in a glycolate-producing state for weeks up to months and substantial product yields were obtained, giving this approach the potential to become a large-scale technology. However, the approach required the addition of a carbonic anhydrase inhibitor (EZA), which makes downstream processing difficult and partly impossible. As an alternative for an inhibitor-free approach, a double mutant strain with defects in *CIA5* and *GYD* was developed by crossing several mutants and wild types.

Ideally, the crossing strategy should result in a strain with high productivity, so large-scale application will be economically feasible. For photosynthetic organisms, biomass productivity is mainly dependent on their photosynthetic efficiency (Vecchi et al. 2020). As phototrophic biomass production goes along with oxygen evolution, the latter can be utilized as an online parameter to monitor cell growth and productivity inside bioreactors (Tamburic et al. 2018). Under light-saturating conditions, the carboxylation reaction of RubisCO becomes the rate-limiting step in photosynthesis. Hence, during the screening process, the maximum photosynthetic capacity P_max_, measured as the maximum oxygen evolution at saturating irradiance, was taken as an indicator of the cell’s biochemical capacity for carbon fixation. Strains with high P_max_-values should be efficient in inorganic carbon assimilation which is reflected in high phototrophic growth rates and thus increased biomass formation. Indeed, screening across the different generations revealed a positive correlation between P_max_ and chlorophyll-based growth rates. The crossing strategy resulted in the double mutant strain D6 which showed both P_max_ and growth rates comparable to the wild types. In biotechnological application, such high growth rates would be an advantage to quickly achieve the initial biomass concentrations required for the glycolate production phase.

For the measurements of short-term glycolate production rates, the inhibitor EZA was added to suppress the function of carbonic anhydrases, similar to the effects of a potential *CIA5* mutation. Thus, glycolate production rates can be compared between different strains regardless of the state of their *CIA5* gene. Under these conditions, high production rates indicate strains with a physiological background that is well adapted for glycolate production. As one of the first products of the photorespiratory pathway, glycolate formation is also dependent on carbon assimilation. Thus, a photosynthetic capacity P_max_ generally corresponds with higher glycolate production rates. The wild-type-like production rates of K14 *gyd* are particularly interesting, as they were measured in a physiological state in which both the C2 cycle was blocked along with a suppression of carbonic anhydrases due to the inhibitor. Thus, as expected, without the inhibitor, glycolate production rates were much lower in the single mutant strain. These results support the feasibility of a *cia5 gyd* double mutant with high glycolate excretion rates in which the C2 cycle and the CCMs are continuously blocked.

However, none of the three isolated double mutants was able to reach the potential of glycolate accumulation established by the wild types. This might simply be due to the low number of double mutants isolated, whose glycolate formation rates then only represent a fraction of the total range due to the low genetic variability between strains. Thus, a certain variability has to be expected among the progeny of crosses, which is also reflected in the glycolate excretion rates of the three double mutant strains. Although a general correlation between P_max_ and glycolate production rates was found, this relationship does not seem to be strict, as can be seen when comparing D4 and D6. While D4 had a 1.2-fold higher P_max_, glycolate production rates were 0.6-fold lower than those of D6. Even though glycolate is a relatively early product of photosynthesis, the yield of excreted glycolate achieved by microalgal cultures is a polygenic trait (Dellero et al. 2016). Accordingly, excretion rates depend on *CIA5*/*GYD* activity, the efficiency of the photosynthetic light reactions (see above), the C2 cycle, and the capacity of cellular glycolate transport. The latter determines how efficiently glycolate can be exported from the cell, but is not yet fully understood. Two transporters for export from the chloroplast have been identified in higher plants: plastidic glycolate/glycerate translocator (PLGG1), a glycolate/glycerate antiporter (Pick et al. 2013; Cui et al. 2021), and BASS6, a transporter from the bile acid sodium symporter (South et al. 2017). Both transporters are located inside the chloroplast envelope. However, transporters for glycolate that export across the plasma membrane are still unknown. Identification of these transporters could help to further optimize glycolate excretion if key components of transport are specifically targeted and improved. However, targeted manipulation of single genes is not always successful, especially with complex traits. Even if product accumulation can theoretically be enhanced, complex interactions and homeostatic feedback between metabolic pathways may lead to undesired outcomes. One such example would be a reduced growth rate, as observed in many industrial microalgae strains with increased lipid accumulation (Courchesne et al. 2009). Thus, these strategies rarely result in significant overall improvement of microalgal production platforms.

For a successful technical application, a suitable strain needs further traits in addition to high glycolate production rates, such as robustness against shear stress which is more common in large-scale reactors due to the required intense mixing or gassing with higher flow rates (Leupold et al. 2013; Wang and Lan 2018). Other important traits include the existence of cellular acclimation strategies that provide tolerance to stress conditions such as high light intensities, extreme temperatures, or nutrient deficiency. Acclimation mechanisms are especially important if cells are kept under frequently changing conditions. This is the case in outdoor reactors with fluctuating light and temperature conditions, but also if the reactor is exposed to periodic dilution and thus internal changes in biomass and nutrient distribution (e.g. due to harvesting). As most acclimation strategies rely on a complex regulation and cooperation of different cellular pathways (Lu et al. 2022), it is likewise difficult to optimize them by targeting single genes. Under these conditions, breeding and successive phenotypic screening might serve as a helpful tool for strain optimization and complement a more targeted approach of genetic engineering.

This study shows that strains can not only be bred and selected in regard to a single gene e.g. the presence of a defective *GYD* or *CIA5* gene, but also regarding complex traits, such as the ability for fast growth or efficient glycolate excretion, which can likewise be inherited during sexual crossing and preserved in subsequent generations. Improving *Chlamydomonas* strains via breeding can therefore be an advantageous strategy if aiming for a complex phenotype.

### The new mutant D6 cia5 gyd can be applied as a simplified glycolate production platform

Blocking of the two metabolic pathways in the double mutant, CCMs and C2 cycle, resulted in new possibilities in biotechnological process control. Based on the altered physiology of the double mutant, the glycolate production system could be simplified in comparison to the wild-type system. For the wild type, glycolate production is not feasible without a CCM inhibitor. It has been reported that induction of CCMs in wild types can be observed within 180 min after switching to low CO_2_ (Brueggeman et al. 2012). Therefore, the expression of carbonic anhydrases leads to increased carbon influx and the subsequent suppression of the glycolate pathway. Consequently, carbon flux is directed into biomass formation instead of glycolate production. This distribution is reversed when the carbonic anhydrase inhibitor is added. Moreover, aeration with 0.2% CO_2_ (A4 condition) is required for glycolate production, since the low CO_2_ concentration in ambient air in combination with the inhibitor led to a collapse of the culture, suggesting carbon deficiency. The amount of dissolved CO_2_ was therefore not sufficient to maintain cell metabolism by passive diffusion alone. It can be assumed that the Calvin cycle cannot provide RuBP in sufficient amounts for glycolate formation. Hence, it can be concluded that under aeration with ambient air the wild type is dependent on active carbon uptake by carbonic anhydrases.

In contrast, glycolate production in the mutant strain D6 was possible without the addition of the inhibitor. The aeration of this system and thus the CO_2_:O_2_ ratio differed from the previously established wild-type conditions. While cultures of D6 collapsed shortly after switching to A3 conditions, they could be kept stable for several weeks under ambient air without a decrease in production efficiency. Considering that cells in ambient air were exposed to a lower CO_2_:O_2_ ratio than in the A3 condition and also a lower total CO_2_ concentration, it is likely that cell death in the A3 condition was not due to carbon deficiency but a result of oxygen accumulation inside the reactor. In contrast to the wild type, D6 was seemingly unable to tolerate these high O_2_ concentrations for longer periods which may point to a potential role of CIA5 and the CCM in the regulation of responses to oxygen stress (Santhanagopalan et al. 2021). The detrimental effects of O_2_ accumulation in closed bioreactors have been observed before (Peng et al. 2017) and can be attributed to the formation of reactive oxygen species (ROS) that damage the photosynthetic apparatus, vital enzymes, membrane structures, and DNA. Resilience to these stress conditions can vary between different strains (Peng et al. 2013), which could explain the different reactions of wild type and the D6 mutant to the oxygen-enriched A3 condition.

Although the daily glycolate production rates of the wild type were two times higher than with D6, this required aeration with a 5-times higher CO_2_ supply than in the D6 cultivation with ambient air. However, for algal production facilities, CO_2_ supply is a major cost factor and an environmental burden for sustainable cultivation. For example, atmospheric CO_2_ levels for biomass-based carbohydrate production from microalgae are not high enough to achieve the growth and productivity rates required for a large-scale production facility (Kumar et al. 2010; Sydney et al. 2010). Enriching the gas stream with compressed CO_2_ in turn increases the economic costs of a facility (Slade and Bauen 2013) as well as its environmental impact since recapturing the surplus CO_2_ outgassed by the reactor is not always feasible (Acién Fernández et al. 2012; Lam et al. 2012). In this scenario, an algal strain capable of producing the desired compound, i.e. glycolate, at an ambient CO_2_ level is an excellent candidate for industrial application.

In contrast to the wild type, the culture of the double mutant D6 without inhibitor was able to grow and produce glycolate under ambient air, suggesting a sufficient carbon flux within the cells of the mutant strain. These observations are consistent with the cell growth of CC-2702 *cia5* when cultured under ambient air (Yun et al. 2021). It further indicates that the combined mutations do not merely substitute for the effects of the inhibitor observed in the wild type. Although *CIA5* has been shown to control most of the carbonic anhydrases that contribute to the CCMs (Fang et al. 2012), the knock-out in the double mutant D6 did not produce the same physiological behaviour as a carbonic anhydrase inhibitor does. The reason for this is unclear, and its elucidation is complicated by the fact that wild type and D6 have a different genetic backgrounds due to the crossing steps. In the absence of a *CIA5*-inducible CCM, D6 might react to low CO_2_ concentrations by upregulating additional carbon concentrating components independent of *CIA5*. Hints for a secondary *CIA5*-independent system in *Chlamydomonas* come from the observation that some carbonic anhydrases seem to be upregulated in the *cia5* mutant (Ynalvez et al. 2008; Brueggeman et al. 2012).

Under glycolate-producing conditions, both wild-type and D6 cultures still produced new biomass, suggesting that not all fixed carbon was directed to glycolate production/excretion. Similar results were observed when CC-2702 *cia5* and a *GYD* mutant were grown in batch cultures under ambient air (Kang et al. 2022). The *GYD* mutant still acquired new biomass while simultaneously producing 720 mg l^−1^ glycolate in 20 days. This was equivalent to a production rate of 0.47 µM glycolate day^−1^. In comparison, the D6 double mutant developed in this study reached a production rate of 0.63 µM glycolate day^−1^ under similar conditions, demonstrating the potential of the combined *CIA5*/*GYD* mutations and the optimized physiological background of the new strain. The optimized single mutant strains M5 *cia5* and K14 *gyd* showed high growth rates, high photosynthetic efficiency, and significant glycolate production with the carbonic anhydrase inhibitor. Nevertheless, both strains produced only low amounts of glycolate with ambient air, highlighting the importance of suppressing both key pathways, the CCM and the C2 cycle, for increased glycolate production.

However, Kang et al. (2022) also showed that increasing production rates with the *GYD* mutant was possible by optimizing culture conditions and refining the cultivation system. Taken together, these findings illustrate how optimization of technical parameters needs to go hand in hand with strain optimization when further developing the microalgal glycolate production system. A more sophisticated regulation of cultivation parameters such as temperature, aeration, pH, and light intensity control inside the reactor would affect biomass growth and indirectly gas distribution. Cultures with stable biomass and maximum glycolate excretion should thus be possible (Taubert et al. 2019). Further elucidation of topics such as the cell carbon partitioning, regulation of CCMs as well as glycolate metabolism and transport in *Chlamydomonas* is therefore essential. In the future, components of these pathways might become promising targets for the generation of new strains.

In summary, glycolate production by *Chlamydomonas reinhardtii* on a larger scale was only possible using a wild-type strain under elevated O_2_ and CO_2_ and with the addition of a carbonic anhydrase inhibitor. By crossing *Chlamydomonas* wild-type and mutant strains we obtained a new double mutant with defects in the CCM regulator gene *CIA5* and the glycolate dehydrogenase. This *cia5*/*gyd* double mutant strain D6 could produce glycolate under ambient air and inhibitor-free conditions. Comparing this production system to the wild-type system, 50% of the glycolate production rates were reached while no additional CO_2_ supply was required. Furthermore, the crossing strategy showed that physiologically robust and highly efficient production strains can be generated by combining genetic material from wild type and mutants. The inhibitor-free approach opens new pathways for the downstream processing of glycolate, as the interfering inhibitor contamination is now absent, enabling additional catalytic processes. Thus, the double mutant expands the range of possible applications for a *Chlamydomonas*-based glycolate production platform, showing that the combination of crossing and transgenesis is a valuable tool for the generation of industrial production strains.

## Supplementary Information

Below is the link to the electronic supplementary material.

Supplementary file1 (PDF 431 KB)

## Data Availability

The datasets generated during and/or analysed during the current study are available from the corresponding author on reasonable request.
