# Supplementary material for: Crossing and selection of Chlamydomonas reinhardtii strains for biotechnological glycolate production
Source: Appl Microbiol Biotechnol. 2022 May 5;106(9-10):3539–54. doi: 10.1007/s00253-022-11933-y (PMC9151519; doi:10.1007/s00253-022-11933-y)
Supplement: Supplementary file 1 — Supplementary file1 (PDF 431 KB) [file 253_2022_11933_MOESM1_ESM.pdf]

## SUPPLEMENTARY DATA

**Journal of Applied Microbiology and Biotechnology**

# Crossing and selection of *Chlamydomonas reinhardtii* strains for biotechnological glycolate production

**Authors:** Antonia Schad[1], Sonja Rössler[1], Raimund Nagel[2], Heiko Wagner[1], Christian Wilhelm[1]

[1] University of Leipzig, Faculty of Life Science, Department of Algal Biotechnology, D-04318 Leipzig, Permoserstraße 15

[2] University of Leipzig, Faculty of Life Science, Department of Plant Physiology, D-04103, Leipzig, Johannisallee 21-23

Table S1: Primer sequence for amplification of the *GYD* and *CIA5* genes

| **Primer** | **Sequence** |
|---|---|
| *GYD* 5'UTR fwd 1 | 5’-GGG TCT CGA CGT AGA AAT GTA GC -3’ |
| *GYD* Jonikas rev1 | 5‘-AGC ATC GTC CGT AGT AAG GC -3‘ |
| *CIA5* Forward | 5’-CTT GTT CTG CGC CTG CCT AGG-3’ |
| *CIA5* Reverse | 5‘-CTC ACG CGA TTG TCC GAG AAT TGC -3‘ |

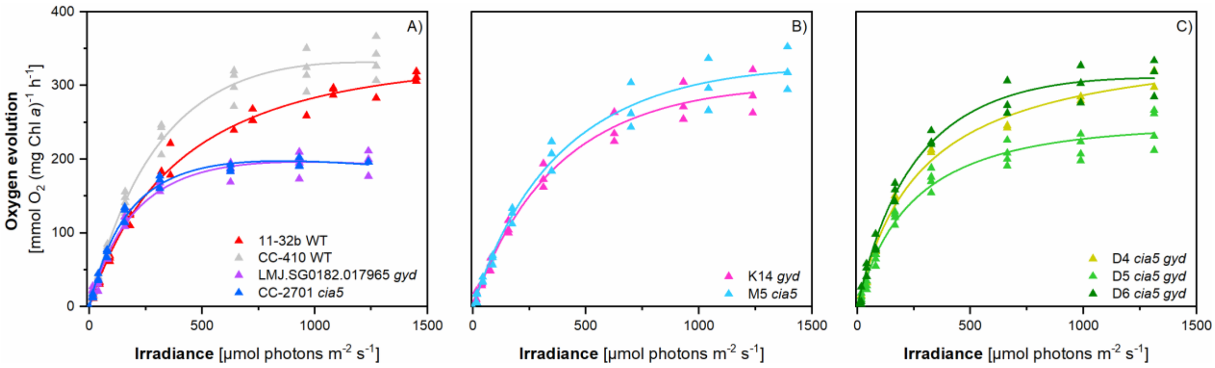


**Figure S1** Gross photosynthesis rates vs. irradiance for *Chlamydomonas* strains of different generations. A): First generation wild types and mutants. B): Second-generation mutants. C): Third-generation double mutants

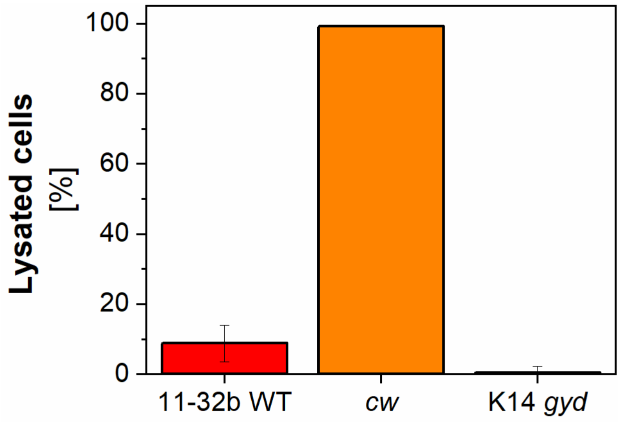


**Figure S2** Percentage of lysed cells after incubating wild type (11-32b WT), a cell-wall less control strain (*cw*) and the new K-line mutant K14 *gyd* with the chemical detergent Triton X-100 (1% (v/v)). Shown are mean values for each strain (n = 3). Error bars indicate standard deviation

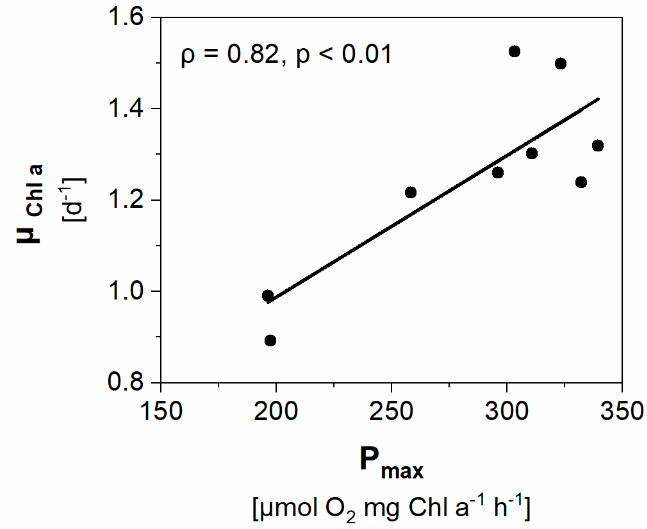


**Figure S3** Correlation between maximum photosynthesis rates $P_{max}$ and chlorophyll-based growth rates $\mu_{Chl\ a}$ in 9 different strains of *Chlamydomonas reinhardtii* wild types and mutants. ρ: Pearson's correlation coefficient

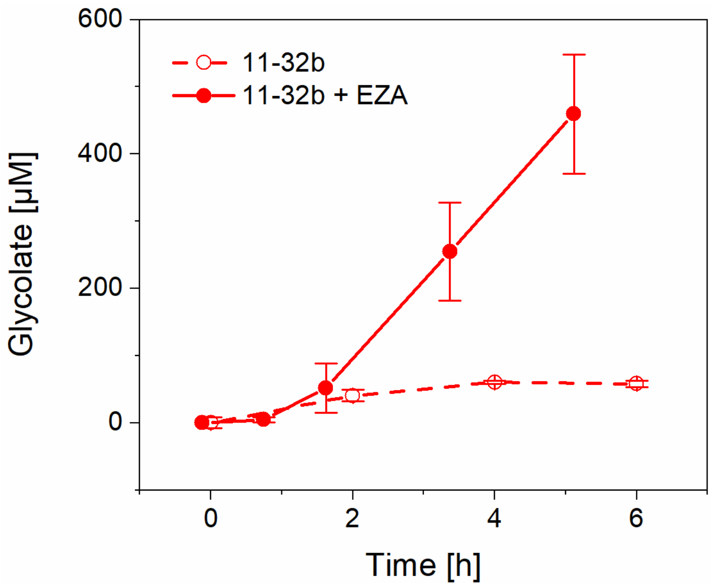


**Figure S4** Short-term glycolate production of wild type 11-32b in inhibitor-free conditions (open dots) or with addition of a carbonic anhydrase inhibitor (closed dots). Cultures were gassed with 40 % $O_2$, 0.08 % $CO_2$ (A3 condition). Shown are mean values for each condition (n = 3). Error bars indicate standard deviation

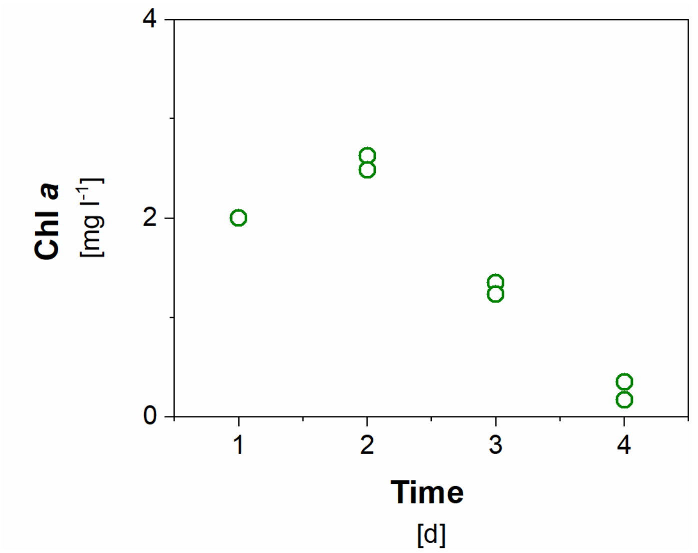


**Figure S5** Chl *a* content of the double mutant D6 when kept on elevated $O_2$ (gassing condition A3 with 40 % $O_2$; 0.08 % $CO_2$) for several days
